# Supplementary material for: Multiple timescales of normalized value coding underlie adaptive choice behavior
Source: Nat Commun. 2018 Aug 10;9:3206. doi: 10.1038/s41467-018-05507-8 (PMC6086888; doi:10.1038/s41467-018-05507-8)
Supplement: Supplementary file 1 — Supplementary Information [file 41467_2018_5507_MOESM1_ESM.pdf]

Supplementary Information

Multiple timescales of normalized value coding underlie adaptive choice behavior

Jan Zimmermann[1], Paul Glimcher[1,2], Kenway Louie[1,2]

[1]Center for Neural Science, New York University, NY, USA

[2]Institute for the Study of Decision Making, New York University, NY, USA

Corresponding author:

Jan Zimmermann jan.zimmermann@nyu.edu

Center for Neural Science

4 Washington Place

10003, New York, NY

Supplementary Discussion

Our modeling results show that adaptive choice behavior at longer timescales can be reproduced by a dynamic normalization circuit, providing a potential circuit mechanism for adaptive value coding. An unresolved question at this time is how slow temporal dynamics are biophysically implemented in neural circuits. One possibility is that these changes result from long term plasticity within decision circuits, akin to proposals for adaptation in the visual system[1]. Another possibility is that pooling the integrated output of value based activity and recurrent feedback may underlie this phenomenon. In our modeling efforts, we purposefully refrained from linking our model stages with particular brain areas; however our estimated slow temporal integration timescales match well with previous electrophysiological studies of adaptation in orbitofrontal cortex and our rapidly adapting stage relates well to data from parietal area LIP[2,3]. However, it is possible that fast and slow timescale functions are served by a number of brain areas; alternatively, both timescales may be part of a single network capable of operating at a range of multiple timescales. Recent evidence of large-scale dynamical models based on connectivity data from tract-tracing experiments suggests a hierarchy of integrative timescales with sensory systems exhibiting brief transient responses and persistent long term activity in associative cortex[4,5]. These findings suggest an unknown circuit mechanism that establishes long temporal receptive windows within prefrontal and temporal areas[6]. One potential explanation for these differences can be regional differences in electrochemical composition of synapses[7]. It is unclear if these synaptic changes are driven primarily within cortical regions or if potential thalamo-cortical projections regulate temporal integration[8], a potential mechanism underlying many theories of learning signals[9]. Mechanistically, we note that our model is more closely aligned to variance adaptation effects[10] than to findings exhibiting range adaptation[11-13].

While the adaptation-induced changes in choice stochasticity in this experiment are relatively small, it is important to consider the small dynamic range in which our animals operate. In total, our animals perform hundreds of trials choosing between relatively low magnitude outcomes; larger reward magnitude variations may drive analogously larger effects in choice behavior. One of the limitations of the current experiment is the block nature of the design, which limits the number of different testable reward environments; future experiments will be required to test whether and how adaptive choice generalizes to different statistical changes representing more complex distributional parameters. It will

also be important to determine whether these adaptation effects generalize over different good categories (i.e. different juice types or good categories). In our block design, neither our experimental animals nor our model required an overt cue or indication of the statistical change in the reward environment. However, it is possible that the monkeys learned to detect changes in environmental statistics and changed their decision behavior between contexts in a top-down manner. We note, however, that such a mechanism could not easily explain the across-session variability in the observed adaptation effect. Our data suggests a very high degree of sensitivity to the precise stochastic sequences of choices offered to the subjects, rather than to the block structure per se. Our shuffle analysis of the reward magnitudes within blocks further supports a continuous, rather than a change point-style process; it also indicates that the precise sequence of rewards and not the general identity of the blocks or statistics are the underlying driver for the adaptation effect we observed.

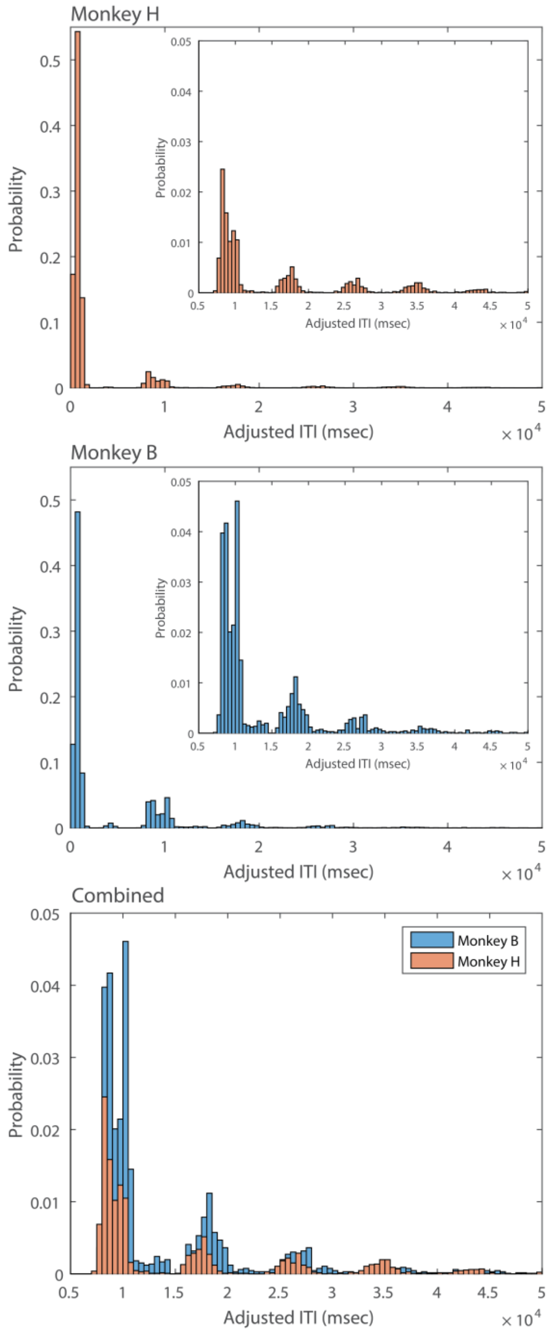

Supplementary Figure 1. Adjusted intertrial interval distributions. Top and middle panels, histograms of the distribution of adjusted intertrial intervals (ITIs) experienced over all testing days in each animal. Adjusted ITIs account for both assigned ITI durations and any timeouts following aborted trials; adjusted ITIs for sequentially aborted trials combine all durations together. Note that the periodicity reflects the timeout duration; inset panels show magnifications of long duration adjusted ITIs associated with aborted trials. Bottom panel, comparison of long duration adjusted ITIs in both animals (same data as insets in top and middle panels). Consecutive aborted trials result in longer durations between successive correct trials. One animal (Monkey B) exhibited more long duration adjusted ITIs.

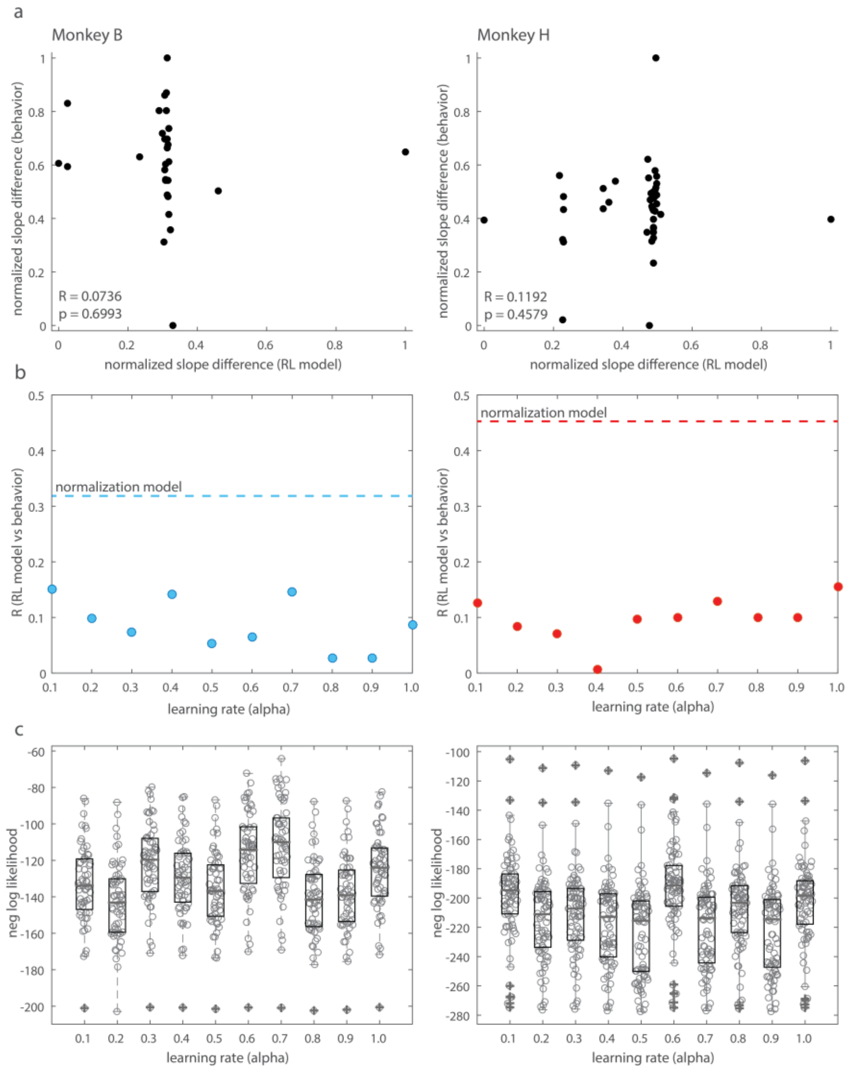

Supplementary Figure 2. Reinforcement learning model results. In this standard RL model, the value of each juice type is learned via prediction error as a function of the sequence of experimental rewards, with choice implemented via a softmax choice function. Two parameters were thus fitted using *fmincon* in Matlab: alpha (the unitary learning rate) as well as beta (the temperature of the softmax function). Parameters were optimized for each individual testing day. The best fitting parameter combination was then used to predict individual choices, and choice curves were fit using the same procedures used in our behavioral and normalization data analyses. (a) Scatterplot of the normalized slope differences obtained from the behavioral data versus the normalized slope differences of the reinforcement learning model. For both animals, learning rate (alpha) as well as softmax inverse temperature (beta) were fit independently for each testing day. (b) Reinforcement learning model performance at different fixed learning rates. Dashed lines, correlation between normalization model predictions and empirical observations of adaptation effects (as shown in Fig. 5 in the main text). None of the RL model correlations are statistically significant (all $p>0.3$). (c) Boxplots of goodness of fit (log likelihood) of the reinforcement learning for fixed learning rates. The center line indicates the median, bottom and top edges of the box indicate the 25th and 75th percentiles, respectively. The whiskers extend to the most extreme data points not considered outliers, and the outliers are plotted individually using the '+' symbol Changing the learning rate does not change the ability of the model to predict monkey choices, suggesting that a reinforcement learning process is not the primary driver of monkey behavior in this task.

Supplementary References

1. Schwartz, O., Hsu, A. & Dayan, P. Space and time in visual context. *Nat. Rev. Neurosci.* **8,** 522–535 (2007).
2. Louie, K., Grattan, L. E. & Glimcher, P. W. Reward value-based gain control: divisive normalization in parietal cortex. *J. Neurosci.* **31,** 10627–10639 (2011).
3. Louie, K., LoFaro, T., Webb, R. & Glimcher, P. W. Dynamic divisive normalization predicts time-varying value coding in decision-related circuits. *J. Neurosci.* **34,** 16046–16057 (2014).
4. Chaudhuri, R., Knoblauch, K., Gariel, M.-A., Kennedy, H. & Wang, X.-J. A Large-Scale Circuit Mechanism for Hierarchical Dynamical Processing in the Primate Cortex. *Neuron* **88,** 419–431 (2015).
5. Chaudhuri, R., Bernacchia, A. & Wang, X.-J. A diversity of localized timescales in network activity. *Elife* **3,** e01239 (2014).
6. Bernacchia, A., Seo, H., Lee, D. & Wang, X.-J. A reservoir of time constants for memory traces in cortical neurons. *Nat. Neurosci.* **14,** 366–372 (2011).
7. Duarte, R., Seeholzer, A., Zilles, K. & Morrison, A. Synaptic patterning and the timescales of cortical dynamics. *Curr. Opin. Neurobiol.* **43,** 156–165 (2017).
8. Mello, G. B. M., Soares, S. & Paton, J. J. A scalable population code for time in the striatum. *Curr. Biol.* **25,** 1113–1122 (2015).
9. Schultz, W., Dayan, P. & Montague, P. R. A Neural Substrate of Prediction and Reward. *Science (New York, N.Y.)* **275,** 1593–1599 (1997).
10. Kobayashi, S., Pinto de Carvalho, O. & Schultz, W. Adaptation of reward sensitivity in orbitofrontal neurons. *J. Neurosci.* **30,** 534–544 (2010).
11. Soltani, A., De Martino, B. & Camerer, C. A range-normalization model of context-dependent choice: a new model and evidence. *PLoS Comput. Biol.* **8,** e1002607 (2012).
12. Rangel, A. & Clithero, J. A. Value normalization in decision making: theory and evidence. *Curr. Opin. Neurobiol.* **22,** 970–981 (2012).
13. Padoa-Schioppa, C. Range-adapting representation of economic value in the orbitofrontal cortex. *J. Neurosci.* **29,** 14004–14014 (2009).
